# Supplementary material for: Post-resolution macrophages shape long-term tissue immunity and integrity in a mouse model of pneumococcal pneumonia
Source: Nat Commun. 2024 May 21;15:4326. doi: 10.1038/s41467-024-48138-y (PMC11109210; doi:10.1038/s41467-024-48138-y)
Supplement: Supplementary file 1 — Supplementary Information [file 41467_2024_48138_MOESM1_ESM.pdf]

Supplementary Figures and Tables.

Post-resolution Macrophages shape long-term tissue immunity and integrity in a mouse model of Streptococcus pneumonia.

Karen T. Feehan[1,7], Hannah E. Bridgewater[1,8], Jan Stenkiewicz-Witeska[1], Roel P. H. De Maeyer[2], John Ferguson[3], Matthias Mack[4], Jeremy Brown[5], Giuseppe Ercoli[5], Connar M Mawer[5], Arne N Akbar[1], James R. W. Glanville[1], Parinaaz Jalali[1], Olivia V. Bracken[1], Anna Nicolaou[6], Alexandra C Kendall[6], Michelle A Sugimoto[1], Derek W Gilroy[1]

[1]Department for Experimental and Translational Medicine, Division of Medicine, 5 University Street, University College London, London WC1E 6JJ, United Kingdom.

[2]Nuffield Department of Orthopaedics, Rheumatology and Musculoskeletal Sciences, Botnar Research Centre, Windmill Road, University of Oxford, OX3 7LD, Oxford, United Kingdom

[3]Translational Science and Experimental Medicine, Research and Early Development, Respiratory and Immunology, BioPharmaceuticals R&D, AstraZeneca, Cambridge, United Kingdom. JF is former employee of AstraZeneca.

[4]Universitätsklinikum Regensburg, Innere Medizin II/Nephrologie-Transplantation, Regensburg, Germany

[5]UCL Respiratory, Division of Medicine, 5 University Street, University College London, London WC1E 6JJ, United Kingdom.

[6]Laboratory for Lipidomics and Lipid Biology, Division of Pharmacy and Optometry, School of Health Sciences, The University of Manchester, Oxford Road, Manchester M13 9PT, United Kingdom

[7]Present address: School of Cancer and Pharmaceutical Sciences, King's College London, London SE1 1UL, United Kingdom.

[8] Present address: Centre for Sports, Exercise and Life Science, Coventry University, Priory St, Coventry CV1 5FB, United Kingdom.

CORRESPONDENCE Derek W Gilroy: d.gilroy@ucl.ac.uk

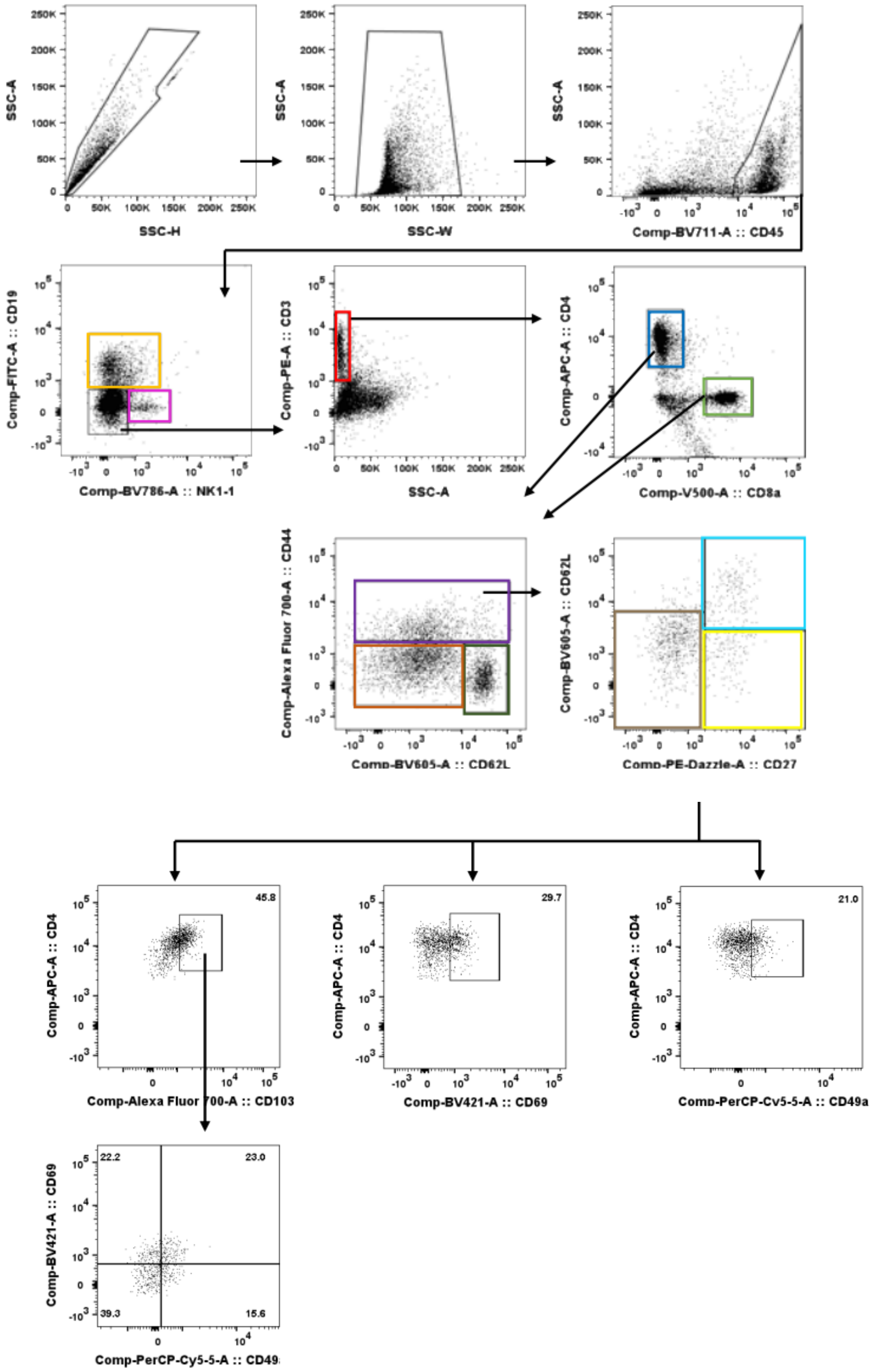

Supplementary Figure 1. Gating strategy to identify T cell populations in the inflamed and resolving mouse lung following inoculation with *S. pneumoniae*.

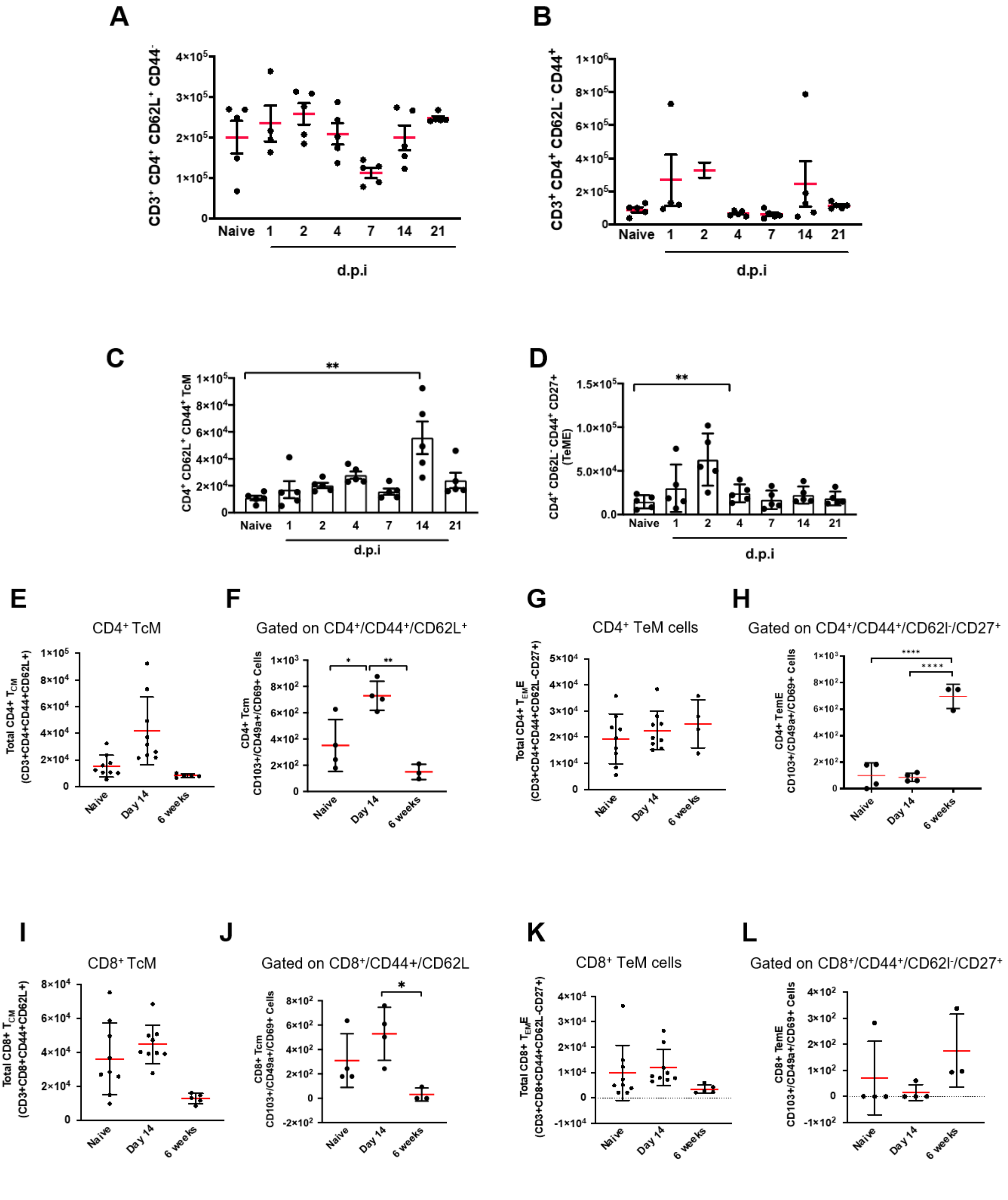

Supplementary Figure 2a. Profiles of T cell populations in the inflamed and resolving mouse lung following inoculation with *S. pneumoniae*.

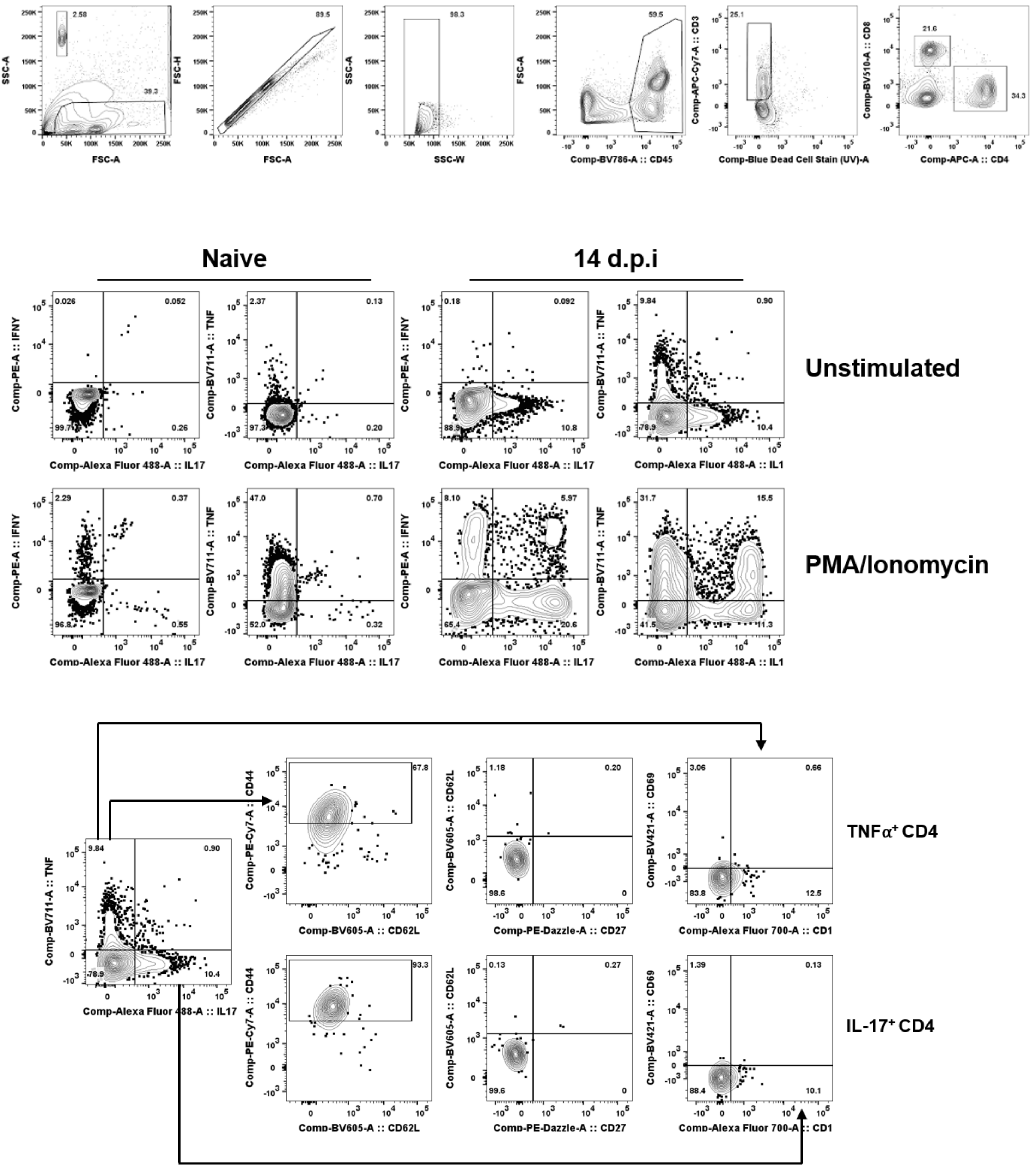

Supplementary Figure 2b. Profiles of T cell intracellular effector molecules in the inflamed and resolving mouse lung following inoculation with *S. pneumoniae*.

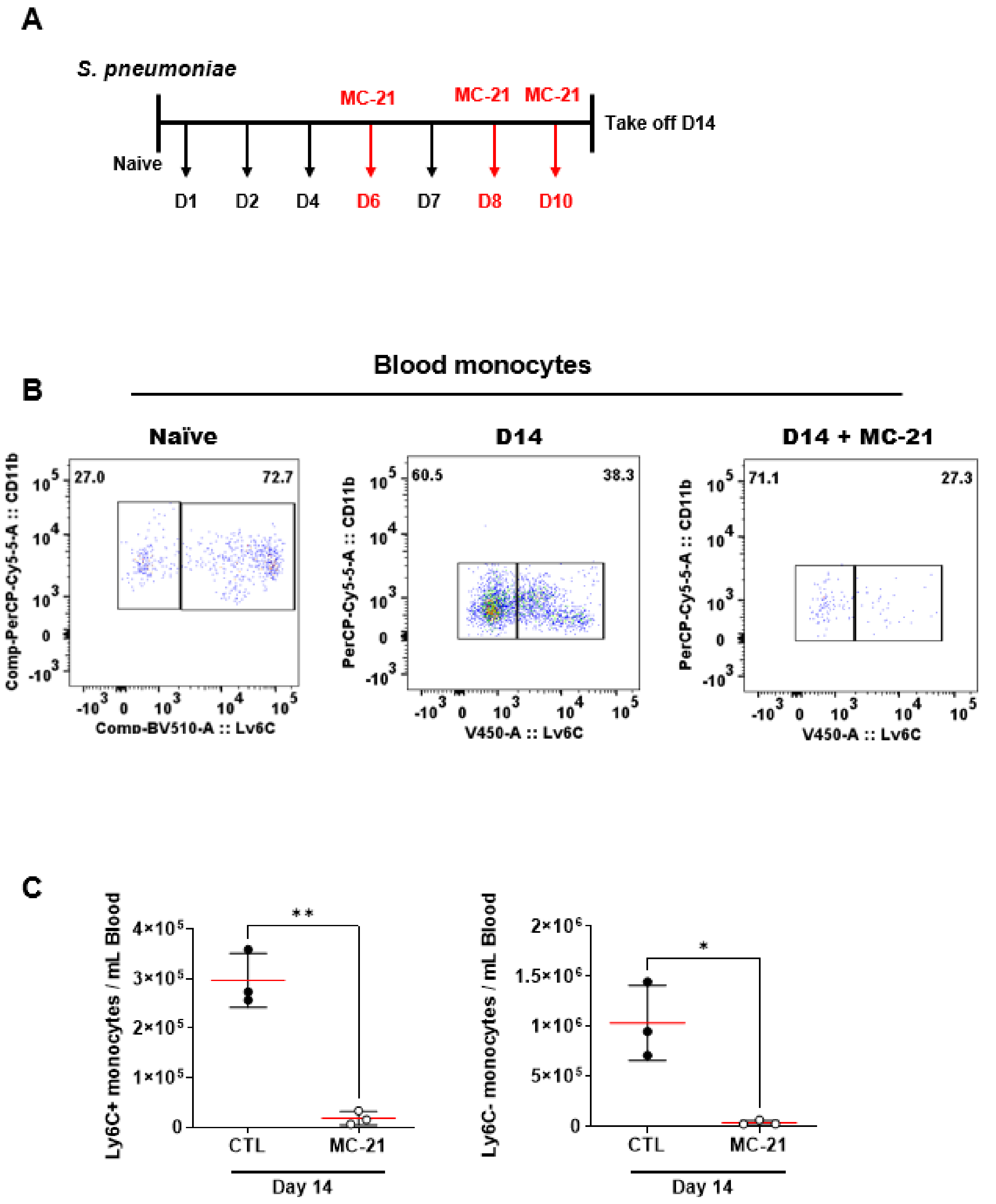

Supplementary Figure 3. Strategy for depleting circulation monocytes in a therapeutic manner in resolving mouse lung following inoculation with *S. pneumoniae* and the impact of this intervention on blood monocyte populations.

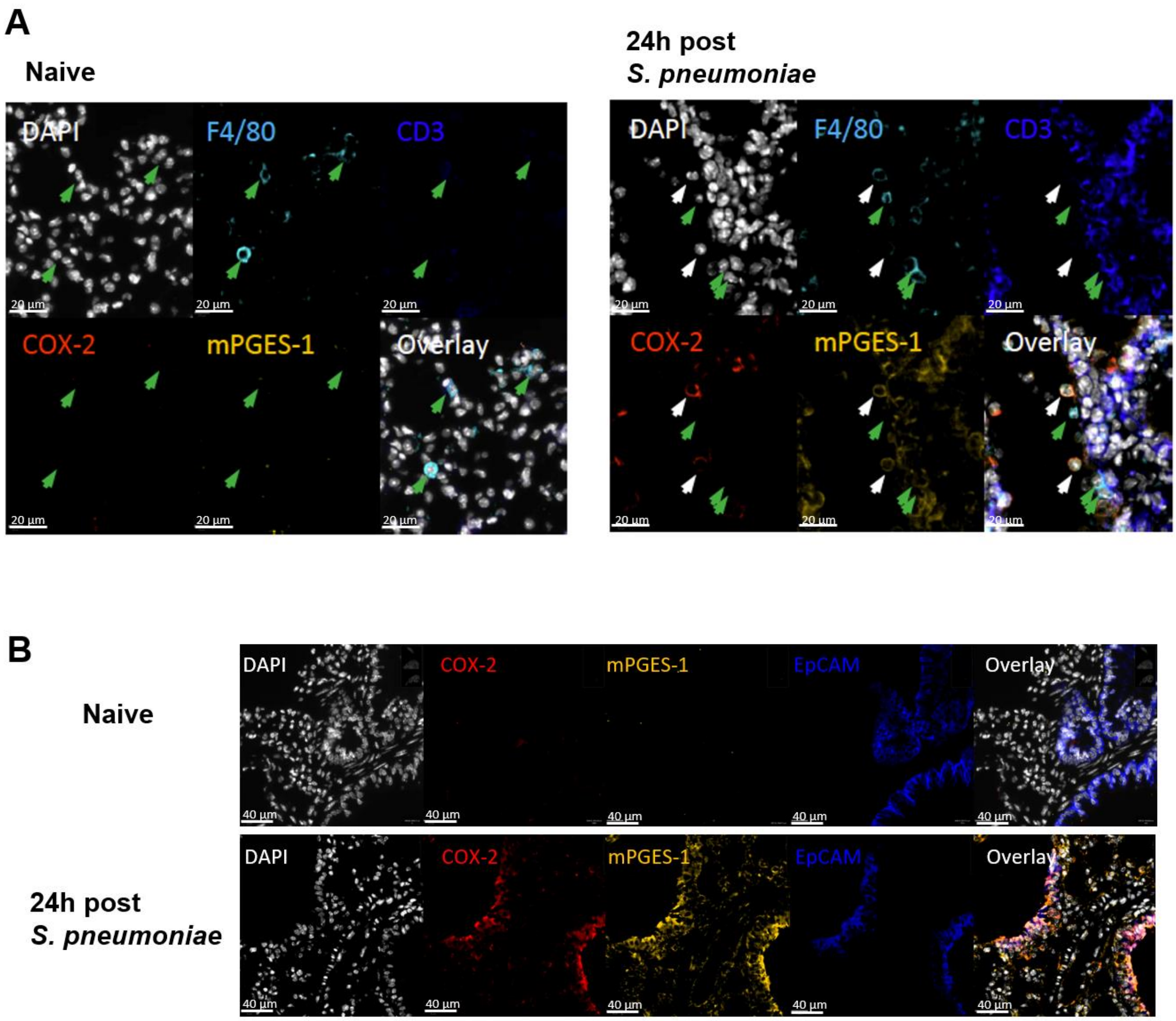

Supplementary Figure 4a. Expression of COX-2 and mPGES-1 at protein level in (A) immune cells and (B) epithelial cells at inflammatory onset following infection with *S. pneumoniae*.

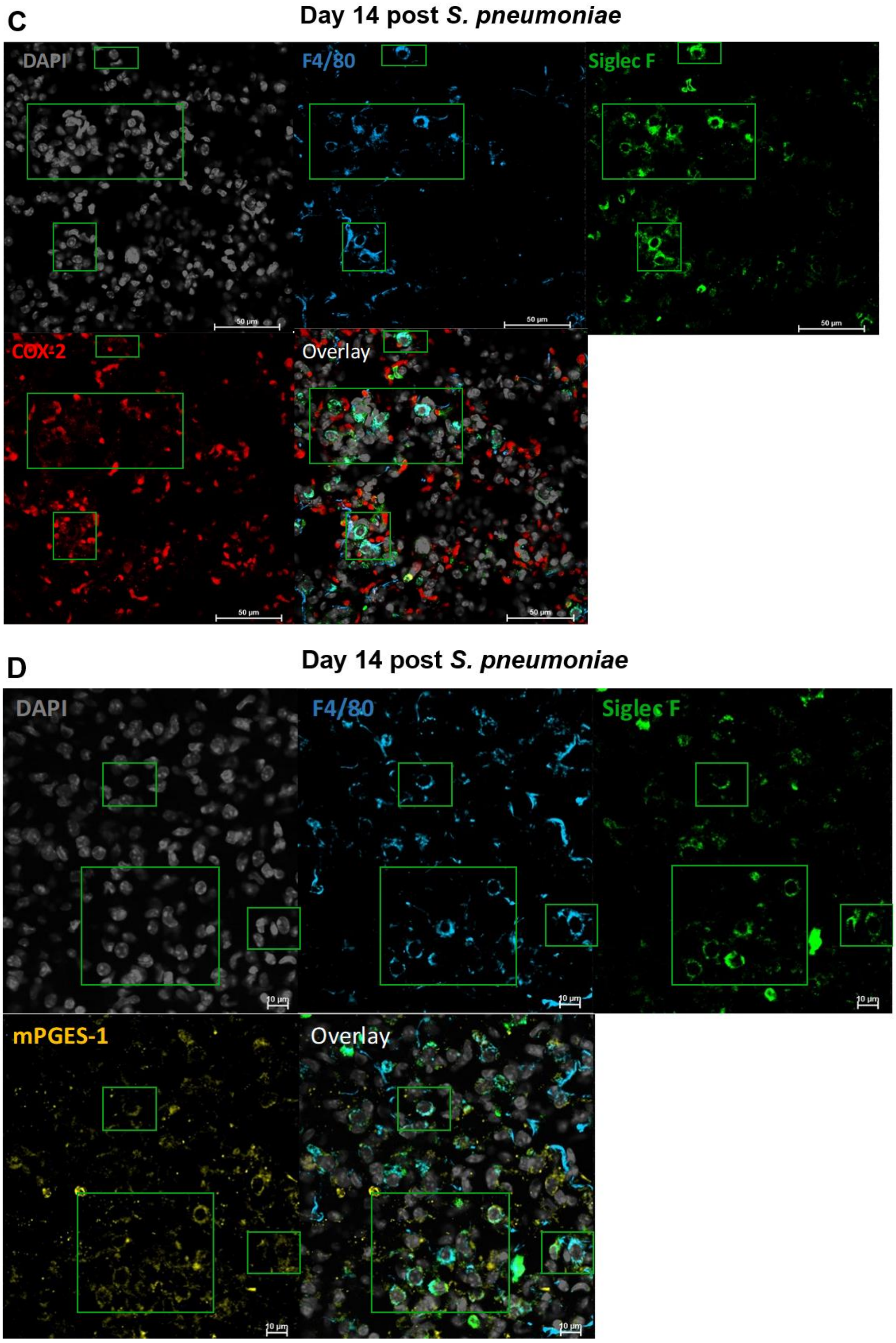

Supplementary Figure 4b. Expression of (C) COX-2 and (D) mPGES-1 at protein level in immune cells at post-resolution phase.

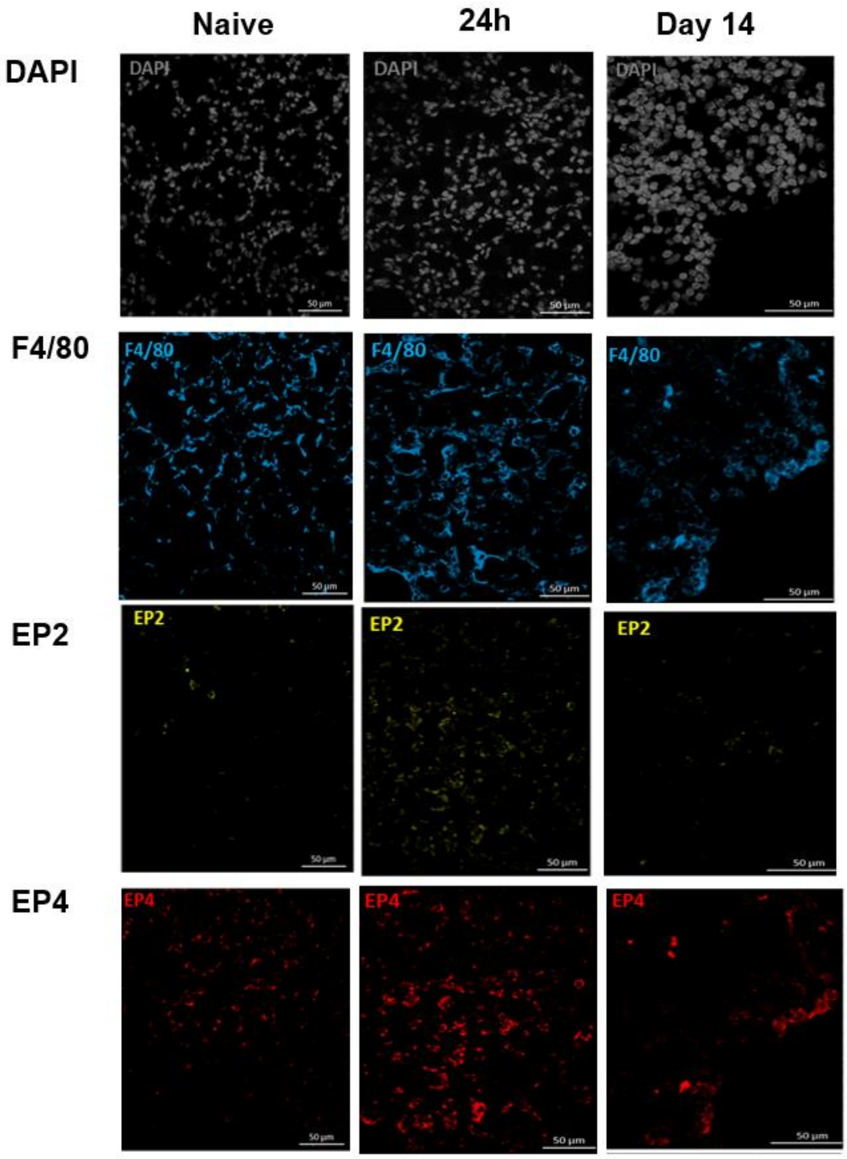

Supplementary Figure 5a. Panels show EP2/4 receptor expression naïve, inflamed, and post-resolved lung. Frozen lung sections from naïve, 24h and 14 days post-infected with *S. pneumonia* mice (n=3) were stained for macrophages (F/480) and assessed for their protein expression of EP2 and EP4.

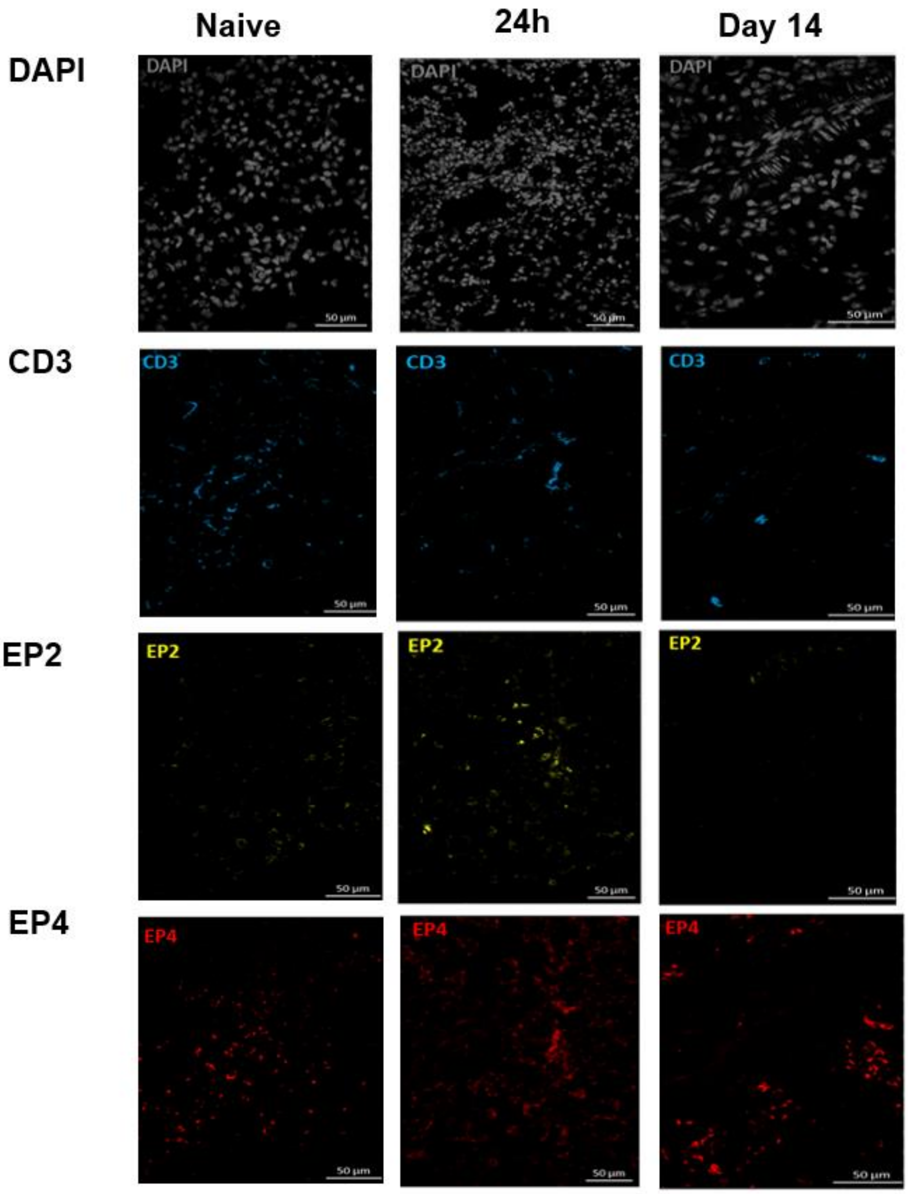

Supplementary Figure 5b. Panels show EP2/4 receptor expression naïve, inflamed and post-resolved lung. Frozen lung sections from naïve, 24h and 14 days post-infected with *S. pneumonia* mice (n=3) were stained for T cells (CD3) and assessed for their protein expression of EP2 and EP4.

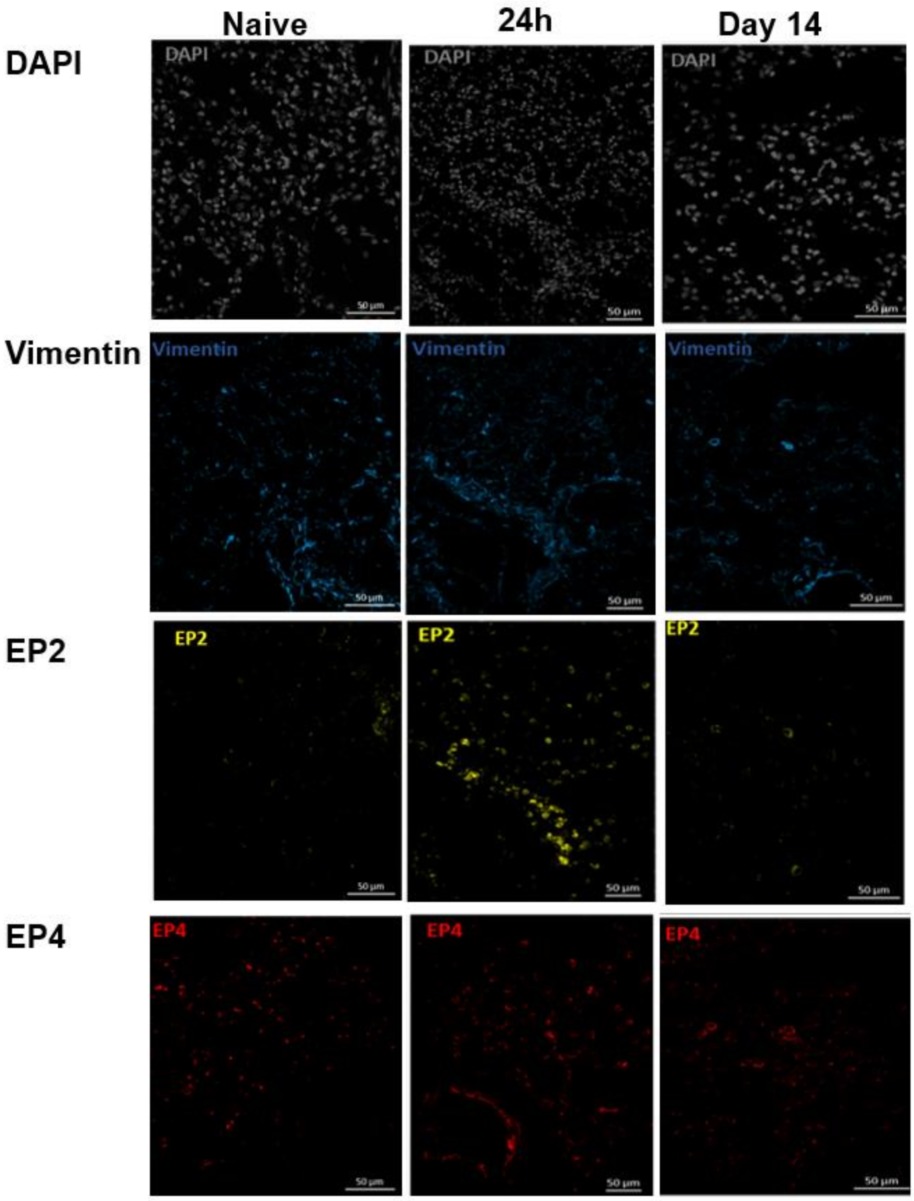

Supplementary Figure 5c. Panels show EP2/4 receptor expression naïve, inflamed and post-resolved lung. Frozen lung sections from naïve, 24h and 14 days post-infected with *S. pneumonia* mice (n=3) were stained for fibroblasts (Vimentin) and assessed for their protein expression of EP2 and EP4.

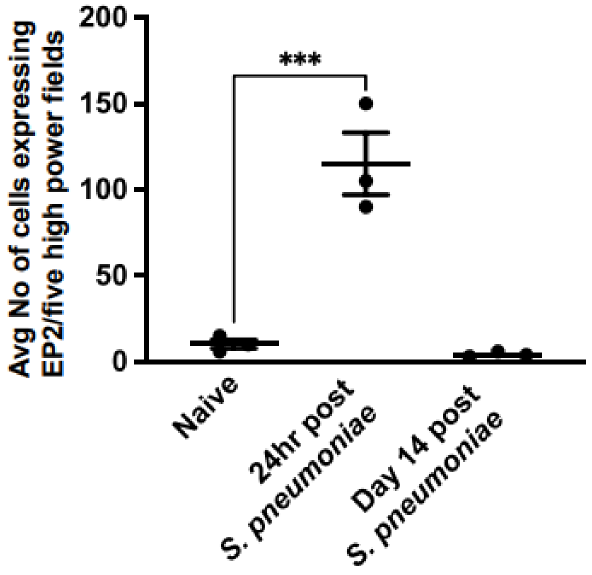

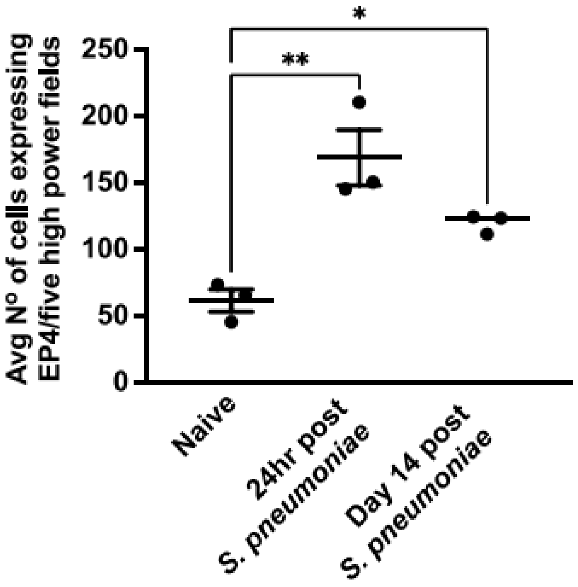

Supplementary Figure 5d. Quantification of immunofluorescence (IF) images showing average number of EP2+ and EP4+ cells in naïve, inflamed, and post-resolved lung.

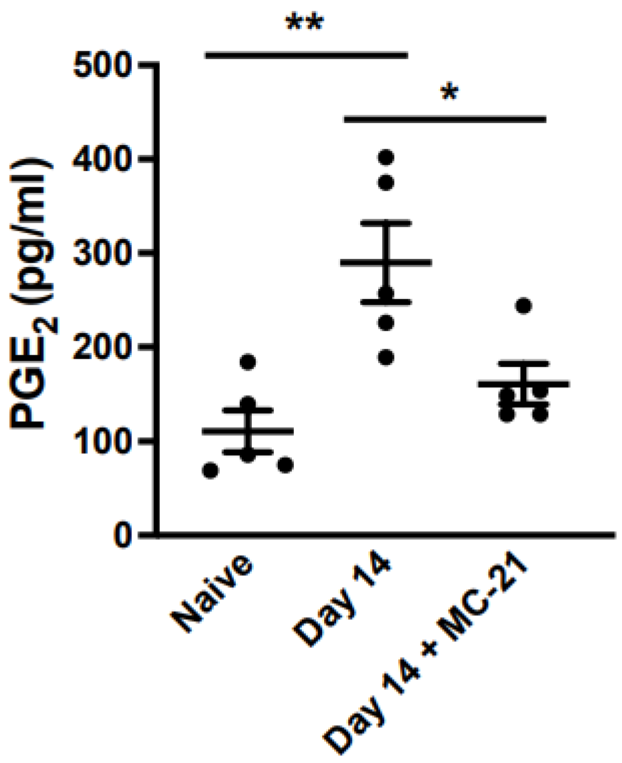

Supplementary Figure 6. Levels of PGE_2 following depletion of lung macrophages using MC-21.

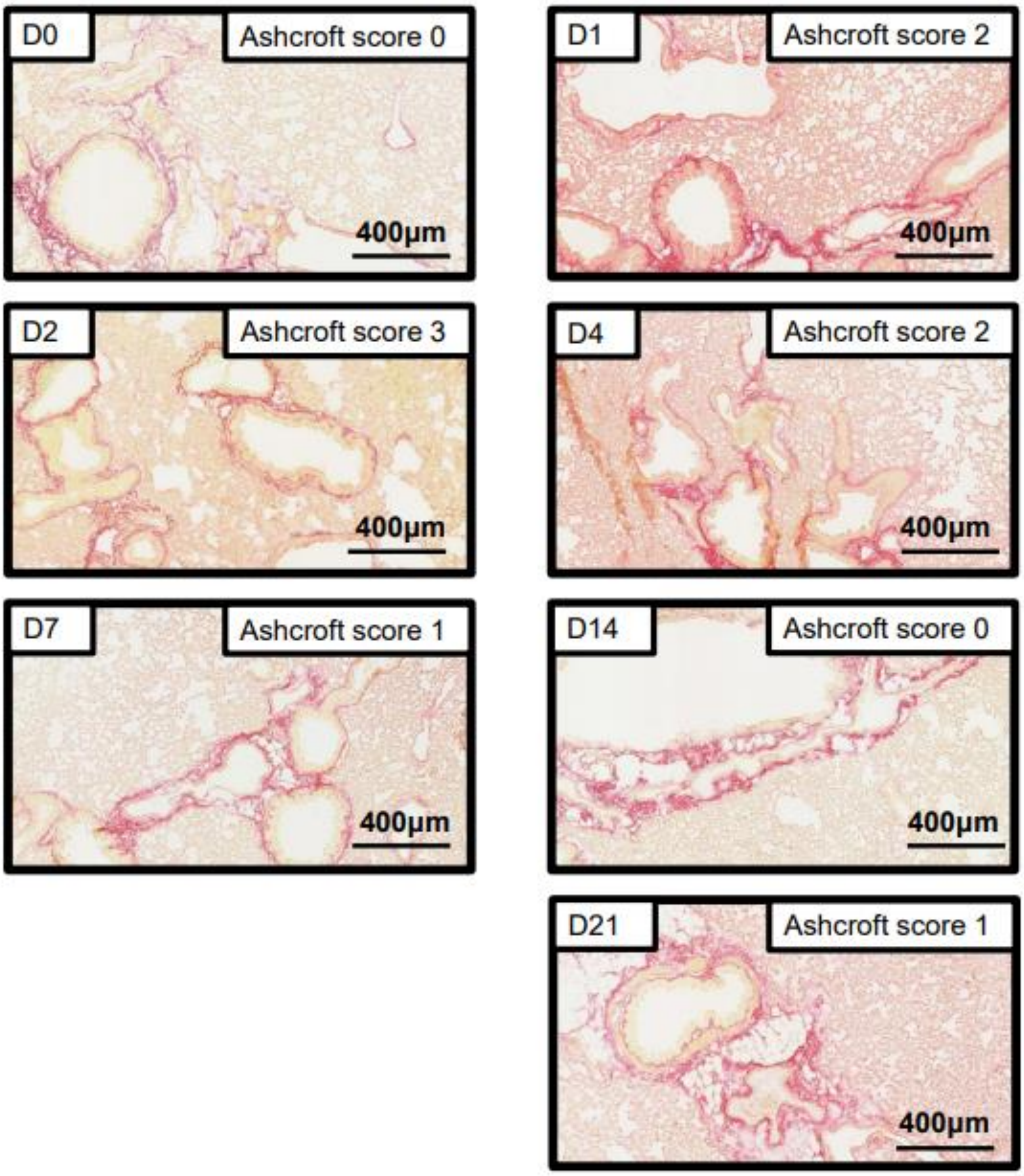

Supplementary Figure 7. Development and resolution of tissue fibrosis in *S. pneumoniae* induced transient lung inflammation.

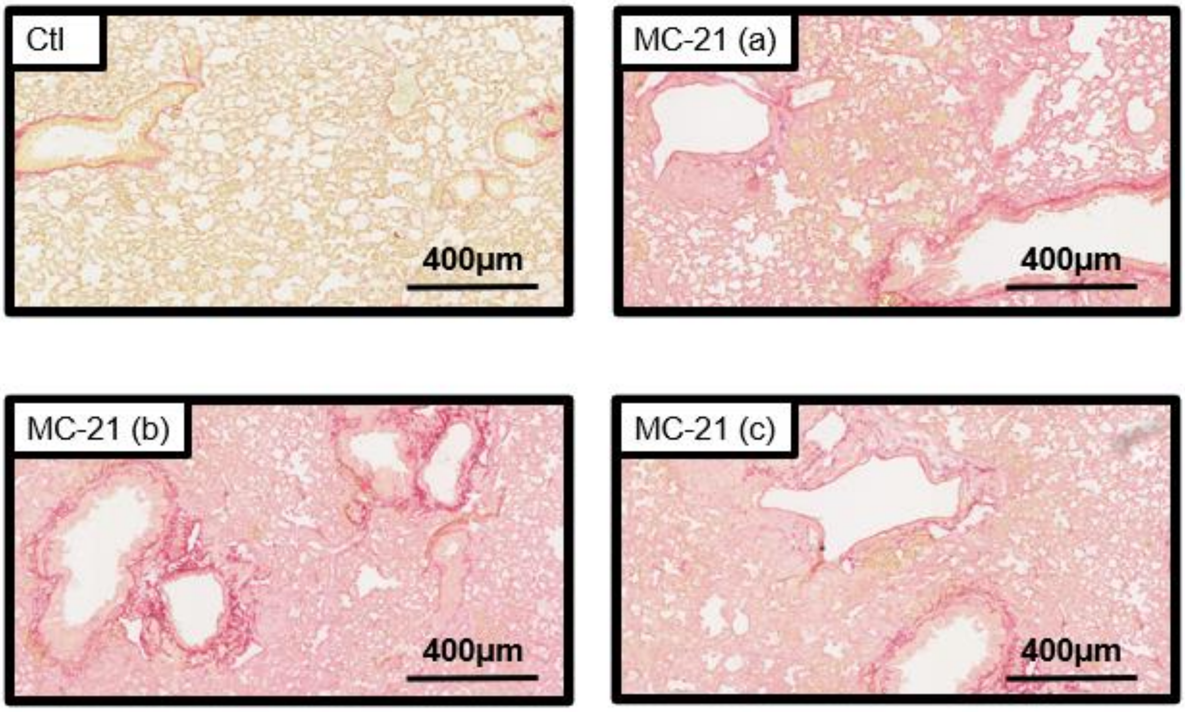

Supplementary Figure 8. Effects of therapeutically depleting post-resolution macrophages with MC-21 on lung fibrosis.

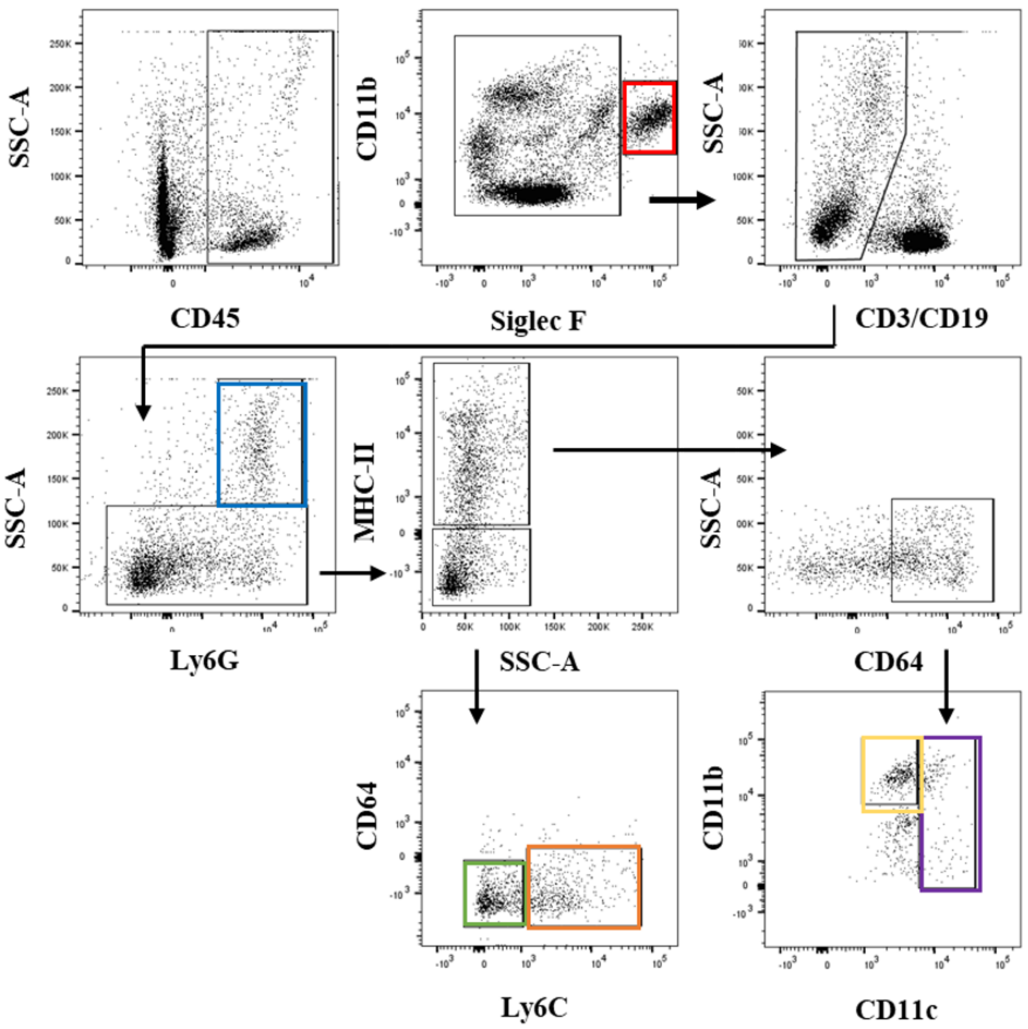

Supplementary Figure 9. Gating strategy to identify myeloid cell population. Immune cells were identified as CD45+ following exclusion of debris and doublets. Alveolar macrophages (Red) were identified as SiglecF^{+} and CD11b^{int}. Neutrophils (blue) were identified as SSC-A^{high} and Ly6G^{+} cells. MHC-II^{+} CD64^{+} cells were further divided into CD11b^{+} CD11c^{-} interstitial macrophage (yellow) and CD11c^{+} dendritic cells (purple). MHC-II^{-} monocytes were subdivided into two populations: Ly6C^{hi} monocytes (orange) and Ly6C^{lo} monocytes (green).

**Gene**	**Sequence**
PTGS2 F	ACACACTCTATCACTGGCACC
PTGS2 R	TTCAGGGAGAAGCGTTTGC
TGFβ F	AGAAGGCAAGCCGGAGGGCA
TGFβ R	ATCCGCTGCTCGGCCACTCT
CYPH F	ATGGTCAACCCCACCGTG
CYPH R	TTCTTGCTGTCTTTGGAACTTTGTC
Il10 F	GGTTGCCAAGCCTTATCGGA
Il10 R	ACCTGCTCCACTGCCTTGCT
IL6 F	CAACGATGATGCACTTGCAGA
IL6R	GTGACTCCAGCTTATCTCTTGGT
PGES F	ATGAGGCTGCGGAAGAAG
PGES R	GCCGAGGAAGAGGAAAGGATAG
EP1F	CAGGGTTCACGCACACG
EP1R	CAGGGAGTTAGAGTTCCAGCC
EP2F	AGACGGACCACCTCATTCTCC
EP2R	GGCCTAAGTATGGCAAAGACCC
EP3F	TGTGTGTGCTGTCCGTCTGTT
EP3R	CTCCTTCTCCTTTCCCATCTGTGT
EP4F	TCTGCTCCATTCCGCTCGT
EP4R	GGCCTGCAAATCTGGGTTTCT
PGISF	CCAGGATGAAGGAAAAGCAC
PGISR	GAAGATGGCATAGGGATGGA
TBXASF	GAAGGTCTGCCGTATCTGGA
TBXASR	TCAGGGTCAAAGGTCTCAGG
CCR2 F	AGCACATGTGGTGAATCCAA
CCR2 R	TGCCATCATAAAGGAGCCA
CCL2 F	ATTGGGATCATCTTGCTGGT
CCL2 R	CCTGCTGTTCACAGTTGCC

Supplementary Table 1. Primer sequences.
