# Supplementary material for: Post-resolution macrophages shape long-term tissue immunity and integrity in a mouse model of pneumococcal pneumonia
Source: Nat Commun. 2024 May 21;15:4326. doi: 10.1038/s41467-024-48138-y (PMC11109210; doi:10.1038/s41467-024-48138-y)
Supplement: Supplementary file 3 — Description of Additional Supplementary Files [file 41467_2024_48138_MOESM3_ESM.pdf]

Description of Additional Supplementary Files

File Name: Supplementary Data 1
Description: Following from Figure 5 In the manuscript, these data are a comprehensive profile of other lipid mediators (prostanoids, hydroxy- and epoxy-fatty acids) in the naïve, inflamed, resolving and post-resolution lung following inoculation with *S. pneumoniae* as measured by LC-MS/MS.
