# Supplementary material for: Post-resolution macrophages shape long-term tissue immunity and integrity in a mouse model of pneumococcal pneumonia
Source: Nat Commun. 2024 May 21;15:4326. doi: 10.1038/s41467-024-48138-y (PMC11109210; doi:10.1038/s41467-024-48138-y)
Supplement: Supplementary file 5 — Reporting Summary [file 41467_2024_48138_MOESM5_ESM.pdf]

nature portfolio

Corresponding author(s): Derek W Gilroy

Last updated by author(s): Feb 7, 2024

Reporting Summary

Nature Portfolio wishes to improve the reproducibility of the work that we publish. This form provides structure for consistency and transparency in reporting. For further information on Nature Portfolio policies, see our Editorial Policies and the Editorial Policy Checklist.

Statistics

For all statistical analyses, confirm that the following items are present in the figure legend, table legend, main text, or Methods section.

n/a	Confirmed	
☐	☒	The exact sample size (*n*) for each experimental group/condition, given as a discrete number and unit of measurement
☒	☐	A statement on whether measurements were taken from distinct samples or whether the same sample was measured repeatedly
☐	☒	The statistical test(s) used AND whether they are one- or two-sided<br>*Only common tests should be described solely by name; describe more complex techniques in the Methods section.*
☐	☒	A description of all covariates tested
☐	☒	A description of any assumptions or corrections, such as tests of normality and adjustment for multiple comparisons
☐	☒	A full description of the statistical parameters including central tendency (e.g. means) or other basic estimates (e.g. regression coefficient) AND variation (e.g. standard deviation) or associated estimates of uncertainty (e.g. confidence intervals)
☒	☐	For null hypothesis testing, the test statistic (e.g. *F*, *t*, *r*) with confidence intervals, effect sizes, degrees of freedom and *P* value noted<br>*Give P values as exact values whenever suitable.*
☒	☐	For Bayesian analysis, information on the choice of priors and Markov chain Monte Carlo settings
☒	☐	For hierarchical and complex designs, identification of the appropriate level for tests and full reporting of outcomes
☒	☐	Estimates of effect sizes (e.g. Cohen's *d*, Pearson's *r*), indicating how they were calculated

Our web collection on statistics for biologists contains articles on many of the points above.

Software and code

Policy information about availability of computer code

Data collection	BD FACSDiva software was used for the collection of Flow cytometry data and for cell sorting experiments.
Data analysis	Graphpad Prism software (Version 8.2.1), FASTQC (Version 0.11.9), HISAT2 (Version 2.1.0), SAMTOOL (Version 1.9) RStudio (Version 1.4.1106), DESeq2 (Version 1.32.0), EdgeR (Version 4.0.5), ggplot (Version 3.3.3), enhanced volcano (Version 1.10.0) PANTHER (Version 16.0), Reactome (Version 76), FlowJo (Version 10.7.2), QuPath (v0.4.4).

For manuscripts utilizing custom algorithms or software that are central to the research but not yet described in published literature, software must be made available to editors and reviewers. We strongly encourage code deposition in a community repository (e.g. GitHub). See the Nature Portfolio guidelines for submitting code & software for further information.

Data

Policy information about availability of data

All manuscripts must include a data availability statement. This statement should provide the following information, where applicable:

- Accession codes, unique identifiers, or web links for publicly available datasets
- A description of any restrictions on data availability
- For clinical datasets or third party data, please ensure that the statement adheres to our policy

RNAseq data was aligned to the publicly available reference mouse genome (GRCm38). Data presented in this paper (RNAseq/lipidomics) are in the process of being deposited to Zenodo (provisional link 10.5281/zenodo.10611080)

Human research participants

Policy information about studies involving human research participants and Sex and Gender in Research.

Reporting on sex and gender	N/A
Population characteristics	N/A
Recruitment	N/A
Ethics oversight	N/A

Note that full information on the approval of the study protocol must also be provided in the manuscript.

Field-specific reporting

Please select the one below that is the best fit for your research. If you are not sure, read the appropriate sections before making your selection.

☒ Life sciences ☐ Behavioural & social sciences ☐ Ecological, evolutionary & environmental sciences

For a reference copy of the document with all sections, see nature.com/documents/nr-reporting-summary-flat.pdf

Life sciences study design

All studies must disclose on these points even when the disclosure is negative.

Sample size	Experiments were designed with advice from a statistician using power analysis to use the minimum number of mice per group to detect significance differences. Group comparisons were made with ANOVA, and between specific groups by unpaired t-test. Skewed data would be logarithmically transformed if necessary. We used historical data to inform on power calculations. Based on a paired t-test, our experiments will have >80% power (lowest 81.9%) to detect the expected mean difference between groups at the 5% significance level. The estimated magnitude of differences between groups and standard errors of the mean are based on previous experiments.
Data exclusions	No data was excluded from this study
Replication	To mitigate non-reproducible results, key experiments were performed succesfully more than once. Mass spec and RNAseq data collection was not replicated but each group contained n=5 replicates per group.
Randomization	Allocation of mice to experimental groups was random.
Blinding	RNAseq/Lipidomic analysis - samples were randomly allocated a number following sample collection and data analysis was acquired blinded. Experiments were blinded where practical - Moreover in all cases blinded and unblinded, we routinely take several steps to avoid bias, including (1) mice are housed in cages of 4 mice per cage allowing for social interaction. (2) To avoid circadian rhythm variances, experiments are scheduled at the same time each day.

Reporting for specific materials, systems and methods

We require information from authors about some types of materials, experimental systems and methods used in many studies. Here, indicate whether each material, system or method listed is relevant to your study. If you are not sure if a list item applies to your research, read the appropriate section before selecting a response.

Materials & experimental systems

n/a	Involved in the study	
☐	☒	Antibodies
☒	☐	Eukaryotic cell lines
☒	☐	Palaeontology and archaeology
☐	☒	Animals and other organisms
☒	☐	Clinical data
☒	☐	Dual use research of concern

Methods

n/a	Involved in the study	
☒	☐	ChIP-seq
☐	☒	Flow cytometry
☒	☐	MRI-based neuroimaging

Antibodies

Antibodies used	Extracellular Flow Cytometry (all 1:100 unless otherwise stated): CD3-PE (17A2, Biolegend[100205]), CD4-APC (GK1.5, Biolegend [100411], CD8a- V500 (53.6.7, BD Biosciences [560778]), CD11b-FITC/PerCP-Cy5.5 (M1/70, Biolegend[101205/101227]), CD11c-BV605 (N418, Biolegend[117333]), CD19-FITC/PE (6D5/ID3, Biolegend[115505/115507]), CD24-PE-Cy5 (M1/69, Biolegend[101811]), CD25-BUV395 (PC61,BD Biosciences[564022]), CD27-PE-Dazzle (LG.3A10, Biolegend[124227]), CD44-PE-Cy7 (IM7, Biolegend[103029]), CD45-BV711/V500 (30-F11, Biolegend[103147]/BD Bioesceinces[561487]), CD49a-PerCP-Cy5.5 (HMa1, Biolegend[142611]), CD64-BV421/APC (X54-5/7.1, Biolegend [139309/139305), CD62L-BV605 (MEL-14, Biolegend[104437]), CD69-BV421 (H1.2F3, Biolegend[104527]), CD103-AF700 (2E7, Biolegend[121441]), F4/80-PE (BM8, Biolegend[123109]), Ly6C-BV510/V450 (HK1.4/AL-21,Biolegend[128033];BD Biosciences[56059]), Ly6G-BUV395/PerCP-Cy5.5 (1A8, BD Biosciences [563978];Biolegend[127615]), Lyve-1-PE-Cy7 (ALY7, ThermoFisher[25-0443-82]), MerTK-FITC (2B10C42, Biolegend[151503]), MHC-II-AF700 ((1:75) M5/114.15.2, Biolegend[107621]), NK1.1-BV786 ((1:75)PK136, Biolegend[108749]) and SiglecF-APC/PE-CF594 (S17007L, Biolegend[155507];E50-2440, BD Biosciences [562757]). CD16/32 (Tru-stain FcX™; Biolegend)<br><br>Intracellular Flow cytometry (all 1:100): IL17-FITC (TC11-18H10.1, Biolegend[506907]), TNF-α-BV711 (MP6-XT22, Biolegend[506349]), IFN-γ-PE (XMG1.2, Biolegend[505807])<br><br>Immunofluorescence (IF): Alexa Fluor® 647 anti-mouse F4/80 [(1:100) BM8, Biolegend(123121)], Alexa Fluor® 488 anti-mouse F4/80 [(1:100)BM8, Biolegend], Alexa Fluor® 488 anti-mouse CD3 [(1:100) 17A2, Biolegend(1002120], Alexa Flour® 467 anti-mouse COX-2 [(1:40) EPR3777, Abcam – Conjugated using ThermoFisher labelling kit A20186], CF®555 anti-mouse CD3 [(1:100) SP162, Abcam - Conjugated using Biotium Mix-nStain™ CF® Dye Antibody Labelling Kits], and primary unconjugated antibodies anti-mouse EP4/ PTGER4 [(1:50) 4A2A12, Proteintech] anti-human mPGES-1 [(1:100) Polyclonal, Cayman Chemical 160140] and anti-mouse Siglec F/ CD170 [(1:20) S17007L, Biolegend], Alexa Fluor® 488 rat anti-mouse Vimentin [(1:100) W16220A, BioLegend], Alexa Fluor® 647 rat anti-mouse CD326/EpCAM [(1:100) G8.8, BioLegend]. UnConjugated anti-mouse EP2/PTGER2 [(1:100) EPR8030(B), Abcam].<br><br>Gifted antibodies: anti-CCR2 monoclonal antibody, MC-21 (supplied by Matthias Mack, Department of Internal Medicine, University Hospital Regensburg, Germany).
Validation	All antibodies used in this research are commercially available. Antibodies were validated (using positive and negative controls) and titrated upon receipt based on the manufacturers recommendations.

Animals and other research organisms

Policy information about studies involving animals; ARRIVE guidelines recommended for reporting animal research, and Sex and Gender in Research

Laboratory animals	Wild-type 8-10-week-old male C57BL6/J mice were maintained in accordance with UK Home Office regulations (Project licence P69E3D849) and housed in individual ventilated cages (IVCs) at ambient temperature.
Wild animals	This study did not involve wild animals
Reporting on sex	This research only included male mice. Female mice were contemplated in creating the study design but the choice of male subjects was made due to the complicated nature of female hormone production and the impact this has on immune/inflammatory responses.
Field-collected samples	This study did not include samples collected from the field.
Ethics oversight	Mice were maintained in accordance with UK Home office regulations (Project licence P69E3D849). All procedures were carried out under the UK's Home Office Animals (Scientific Procedures) Act 1986.

Note that full information on the approval of the study protocol must also be provided in the manuscript.

Flow Cytometry

Plots

Confirm that:

☒ The axis labels state the marker and fluorochrome used (e.g. CD4-FITC).

☒ The axis scales are clearly visible. Include numbers along axes only for bottom left plot of group (a 'group' is an analysis of identical markers).

☒ All plots are contour plots with outliers or pseudocolor plots.

☒ A numerical value for number of cells or percentage (with statistics) is provided.

Methodology

Sample preparation	Isolated lungs from C57BL6/J mice were digested in 150 U/mL collagenase type IV (Sigma) supplemented with DNase (1 mg/ mL), at 37°C for 45 minutes before being passed through a Falcon 70 µM cell strainer. Red blood cells were lysed (ACK lysis buffer) and whole lung cells were blocked with anti-mouse CD16/32 (Tru-stain FcX™; Biolegend) then stained with

fluorochrome-conjugated antibodies and fixed (4% PFA) prior to analysis. For intracellular cytokine staining, lung cells (106 cells per well) were seeded in 96-well round bottom plates and stimulated or not with PMA (50 ng/ml)/ionomycin (500 ng/ml) or 105 CFU of heat-killed S. pneumoniae (heated at 65 oC for 30 minutes) for 4-24h. All samples were treated with a protein transport inhibitor containing brefeldin A (Biolegend, 1x) during the final 4 hours of incubation. After stimulation, cells were stained with a viability marker (Zombie UV™, Biolegend), blocked with anti-mouse CD16/32 (Tru-stain FcX™; Biolegend) and stained with fluorochrome-conjugated antibodies against surface markers.
Blood was ACK lysed prior to staining.

Instrument

BD Fortessa UV X20 Flow Cytometer. Cell sorting was performed on FACSAria II

Software

BD FACSDIVA software for sample acquisition and FlowJo software (V10.7.2) for analysis.

Cell population abundance

Cell abundance in post-sort populations was determined using the gating strategy in Figure 2.A. On average alveolar macrophages accounted for ~10% of CD45+ cells. Of the remaining CD64+, F4/80+ and MerTK+ macrophages; Lyve1+MHC-II- accounted for 30%, Lyve1-MHC-II+ accounted for 20%, and Lyve-MHC-II- accounted for 50%. In other flow cytometry experiments absolute cell number was calculated using CountBrightTM Absolute Counting Beads (ThermoFisher Scientific) and the gating strategies described in the paper. Number of cells is normalised to tissue weight.

Gating strategy

Myeloid cells: Immune cells were identified as CD45+ following exclusion of debris and doublets. Alveolar macrophages were identified as SiglecF+ and CD11bint. Neutrophils were identified as SSC-Ahigh and Ly6G+ cells. MHC-II+ CD64+ cells were further divided into CD11b+ CD11c- interstitial macrophages and CD11c+ dendritic cells. MHC-II- monocytes were subdivided into two populations: Ly6Chi monocytes and Ly6Clo monocytes. Gating shown in supplementary information.
T cells: B lymphocytes were identified as CD19+. NK Cells were identified as NK1.1+ cells. All T lymphocytes were identified as CD3+ cells and were further divided into CD4+ T Cells and CD8+ T Cells. Both CD4+ and CD8+ T cells were divided into Naïve T Cells, effector memory T Cells and central Memory T Cells based on their differing expression profile of CD44 and CD62L. Memory T Cells were then further divided into CD62L+, CD27+ TCM, CD62L- CD27+ TEME and CD62L- CD27- TEML. These populations were further analysed using the resident memory markers CD103, CD49a and CD69 as well as the intracellular cytokines IL-17, IFNy and TNFa. Gating shown in supplementary information.

☒ Tick this box to confirm that a figure exemplifying the gating strategy is provided in the Supplementary Information.
